# Mapping of global research output in congenital cataracts from 1903 to 2021

**DOI:** 10.1097/MD.0000000000027756

**Published:** 2021-12-03

**Authors:** Lujain Talaat Idriss, Maryam Hussain, Muhammad Khan, Tauseef Ahmad, Khushi Muhammad, Mukhtiar Baig, Muhammad Mumtaz Khan, Inamullah

**Affiliations:** a Department of Ophthalmology, Faculty of Medicine, Rabigh, King Abdulaziz University, Jeddah, Saudi Arabia; b Department of Biotechnology and Genetic Engineering, Hazara University Mansehra, Mansehra, Khyber Pakhtunkhwa, Islamic Republic of Pakistan; c Department of Epidemiology and Health Statistics, School of Public Health, Southeast University, Nanjing, China; d Department of Clinical Biochemistry, Faculty of Medicine Rabigh, King Abdulaziz University, Jeddah, Saudi Arabia; e Department of Microbiology, University of Haripur, Haripur, Khyber Pakhtunkhwa, Islamic Republic of Pakistan.

**Keywords:** bibliometric analysis, congenital cataract, HistCite^TM^, VOSviewer software, Web of Science

## Abstract

**Background and aim:**

Globally, congenital cataract remains one of the main causes of visual loss in children. This study was designed to plot the overall research output and evaluate some key bibliometric indicators in congenital cataracts research.

**Methods:**

Publications on congenital cataracts were retrieved from the Web of Science Core Collection database. The published literature was searched using the keywords “congenital cataract” OR “congenital cataracts” in the title filed with document types and language restrictions. The data were exported into HistCite to analyze; publication year, top authors, countries, institutions, journals, keywords, and most cited studies. VOSviewer software was used to construct network visualization mapping.

**Results:**

A total of 1427 publications (1903–2021) published in English language were included in this study. Over the past few decades, the total number of publications in congenital cataracts was found to be increased. The most productive year was 2016 (n = 72), while the most cited year was 1941 (1268 citations). The *Investigative Ophthalmology & Visual Science* (Impact Factor: 4.799) was the most attractive journal with 161 publications, and the *Molecular Vision* (Impact Factor : 2.367) was the most cited journal with 1915 citations and 161.723 citations per year. The most productive country was the United States of America (USA) (n = 325), while the most active institute was Sun Yat-sen University, China (n = 36). The most prolific author was Yao K (n = 27). The most studied Web of Science category was ophthalmology (n = 852). The most widely used keyword was congenital (n = 1427). The most cited paper in congenital cataracts was “Congenital cataract following German measles in the mother, cited 1268 times. The USA and author keyword congenital cataract had the highest total link strength.

**Conclusion:**

These findings provide useful insights, current status, and trends in clinical research in congenital cataracts. This study can be used to identify future research areas and standard bibliography references for better diagnosis and disease control.

## 1 Introduction

A congenital cataract is a major cause of reversible blindness in children worldwide. The majority of congenital cataract cases are inherited. According to World Health Organization, globally, 95 million people are visually impaired due to cataracts.^[1]^ There are various genetic, environmental, and metabolic factors are associated with congenital cataracts. Norrie disease, caused by a mutation in the NDP gene and inherited as X-linked recessive patterns, is closely associated with congenital cataracts.^[2,3]^ Another X-linked recessive disorder is Nance Horan syndrome resulted severe bilateral congenital cataracts in which males have congenital nuclear cataracts.^[4]^ Down syndrome is a common chromosomal disorder of chromosome 21, causes mental disability and delayed growth. Children with Down syndrome have a greater risk of ocular abnormalities such as congenital cataracts.^[5]^

Control of childhood blindness is a priority of the World Health Organization global proposal to eradicate avoidable blindness. Due to the development of sequencing technology and stem cell research, congenital cataract screening, and treatment have rapidly improved in the past decade.^[6,7]^ Genetic, metabolic, traumatic, and infectious factors can all lead to childhood cataracts. Among these causes, hereditary cataracts constitute 22.3% of global childhood cataracts.^[8,9]^ Mutation screening of inherited congenital cataracts have identified nearly 200 locus and more than 100 causative genes, which are well summarized in the “Cat-Map” website.^[10–12]^ The candidate gene mappings may provide a deeper perception of the pathological basis for cataracts and the natural lens growth process and physiology. They may be helpful for prenatal diagnosis and genetic counseling.^[13]^

## 2 Rationale and aim

Bibliometric analysis in health sciences and other disciplines is being used to evaluate the development of publication in a specific area of research to identify global research output and trends. This type of analysis permits one to assess the impact and influence of scientific work by tracking citations and other key bibliometric indicators.^[14–20]^ The Web of Science (WoS) is one of the widely used databases for bibliometric analysis.^[21–25]^ Several bibliometric studies have been conducted on ophthalmology and visual sciences in specific countries.^[26,27]^ Therefore, this study was performed to figure out the global research output and plot the published literature on congenital cataracts. This study might be helpful for researchers, physicians, and policymakers to pay special attention to congenital cataracts.

## 3 Methods

### 3.1 Study design

A descriptive bibliometric study was designed.

### 3.2 Study participants

In this study no participants were directly involved as the data were downloaded from online database.

### 3.3 Database and search strategy

An online search was conducted by Tauseef Ahmad on July 27, 2021 through Web of Science Core Collection (WoSCC) database, Science Citation Index Expanded (SCI-EXPANDED) Edition hosted by Clarivate Analytics (https://clarivate.com/webofsciencegroup/solutions/web-of-science/). The WoS database was accessed through Southeast University online library portal (http://www.lib.seu.edu.cn/). The Boolean search strategy was applied using the potential keywords “congenital cataract” OR “congenital cataracts” in the title field with document types and language restrictions.

### 3.4 Data extraction

A self-designed data-sheet was used for data collection. The retrieved results were downloaded in plaintext and comma-separated values format. The following data were extracted; publication year, document type, author names, country, institution, journal, funding agency, WoS category, publisher, keywords, and top-cited publications. The Impact Factor of journals was obtained from Incites Journal Citation Reports (released in June 2021 by Clarivate Analytics).

### 3.5 Data analysis

First, the data were exported to Microsoft Office 2013 to calculate frequencies and percentages and then transferred to OriginPro 2018 (https://www.originlab.com/) to generate relevant graphs and pie charts. Different bibliometric key indicators were analyzed using HistCite^TM^ software (http://www.histcite.com/). The plaintext dataset was then exported in to VOSviewer software version 1.6.16 for windows (https://www.vosviewer.com) to construct network visualization mapping (co-authorship countries and author keywords).

### 3.6 Ethical approval

In the current no animal or human subjects were recruited directly. Therefore, no ethical approval was required.

## 4 Results

### 4.1 Characteristics of global research output on congenital cataract

**Figure 1:**
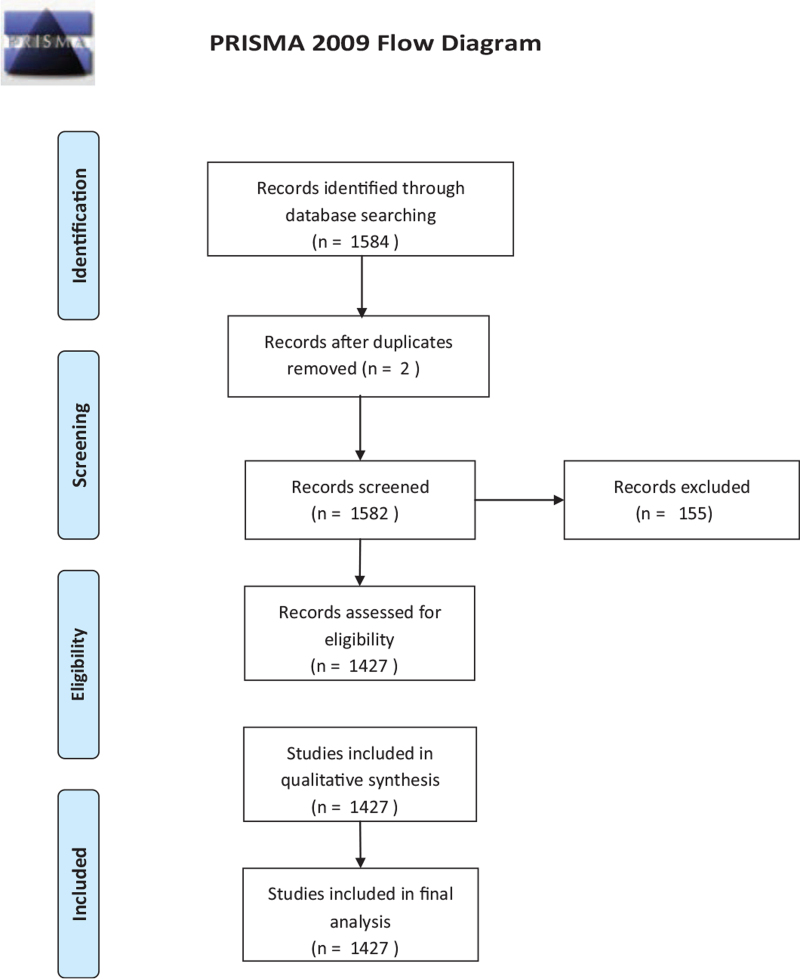
Current study flow chart.

**Figure 2:**
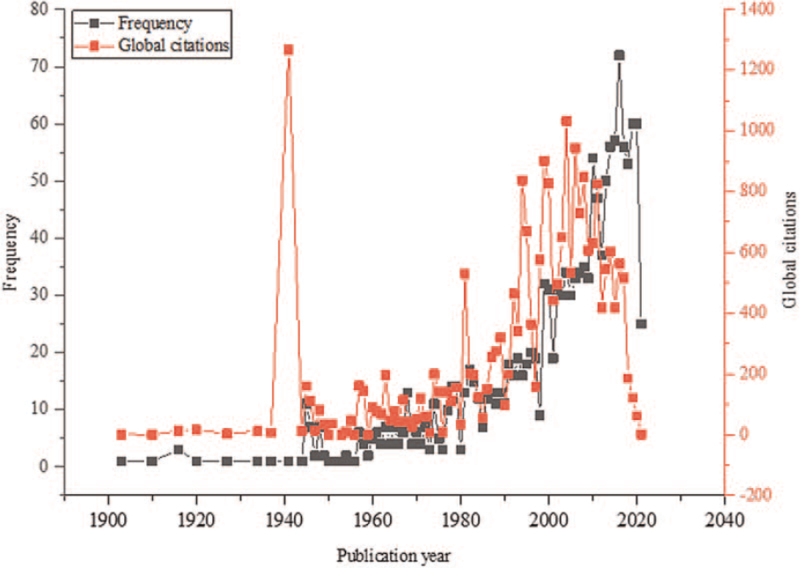
Frequency of publications and global citations from 1903 to July 27, 2021.

A total of 1427 publications on congenital cataracts published from 1903 to 2021 in the English language were included in this study as shown in Figure 1. The included publications were cited 22665 times ranging from 1 to 1268 citations. Over the past few decades, the total number of publications continually increased from 1 in 1903 to 72 in 2016 on congenital cataracts. The most productive year was 2016 (n = 72), while the most cited year was 1941 (1268 citations), as shown in Figure 2.

**Figure 3:**
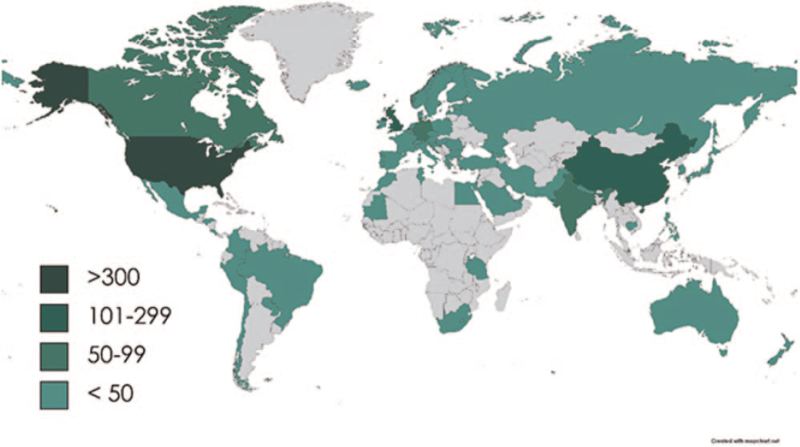
Heat mapping of global research in congenital cataract.

**Figure 4:**
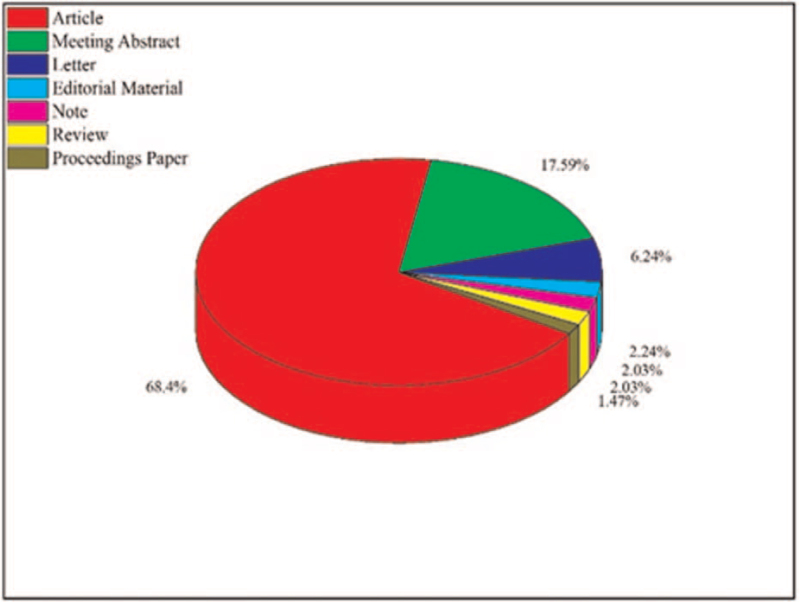
Document types.

The heat mapping of global research in congenital cataracts is presented in Figure 3. The National Natural Science Foundation of China was the leading funding agency in congenital cataracts (n = 134) (Figure S1, Supplemental Digital Content). In congenital cataracts research the top publisher was Elsevier (n = 233) (Figure S2, Supplemental Digital Content). More than 68% (n = 976) publications were articles, and only 2.03% (n = 29) were reviews, as shown in Figure 4.

**Figure 5:**
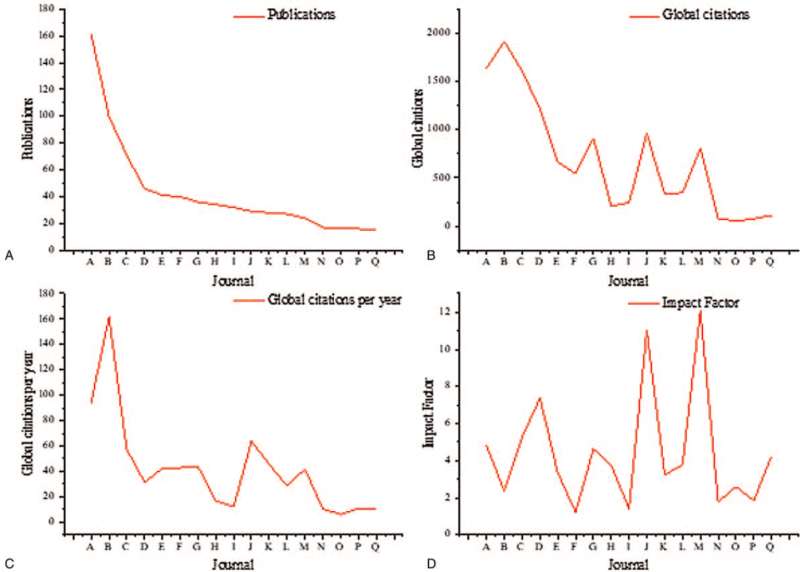
Top journals in congenital cataract research published at least 15 papers. (A) Publications; (B) Global citations; (C) Global citations per year; (D) Journals Impact Factor released in June 2021. **Note:** A: Investigative Ophthalmology & Visual Science; B: Molecular Vision; C: American Journal of Ophthalmology; D: Archives of Ophthalmology (it changed its name to JAMA Ophthalmology in 2013); E: Journal of Cataract and Refractive Surgery; F: Journal of AAPOS; G: British Journal of Ophthalmology; H: Acta Ophthalmologica; I: Journal of Pediatric Ophthalmology & Strabismus; J: American Journal of Human Genetics; K: PLoS One; L: Eye; M: Ophthalmology; N: International Journal of Ophthalmology; O: European Journal of Ophthalmology; P: Indian Journal of Ophthalmology; Q: European Journal of Human Genetics.

**Figure 6:**
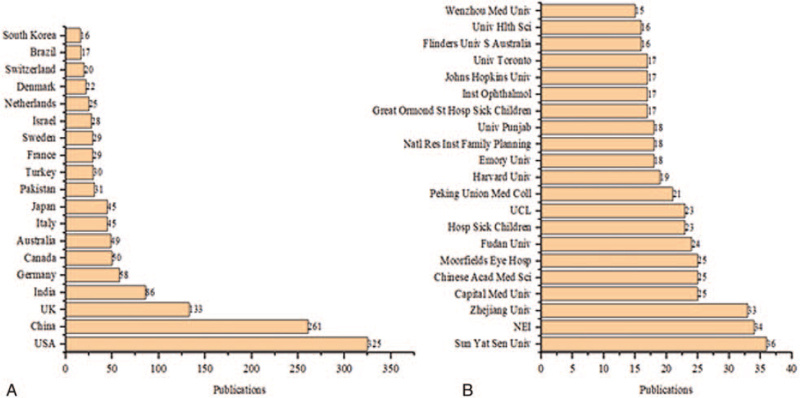
(A) Top countries in congenital cataract research with more than 15 publications. A total of 232 publications were excluded from the above graph based on the missing country name. (B) Top institutes in congenital cataract research with at least 15 publications. A total of 192 publications were excluded from the above graph based on missing institution names.

The *Investigative Ophthalmology & Visual Science* (impact factor: 4.799) was the most attractive journal with 161 publications. The *Molecular Vision* (IF: 2.367) was the most cited journal with 1915 citations and 161.723 citations per year, as shown in Figure 5. The most productive country was the United States of America (USA) (n = 325), while the most active institute was Sun Yat-sen University, China (n = 36), as shown in Figure 6.

**Figure 7:**
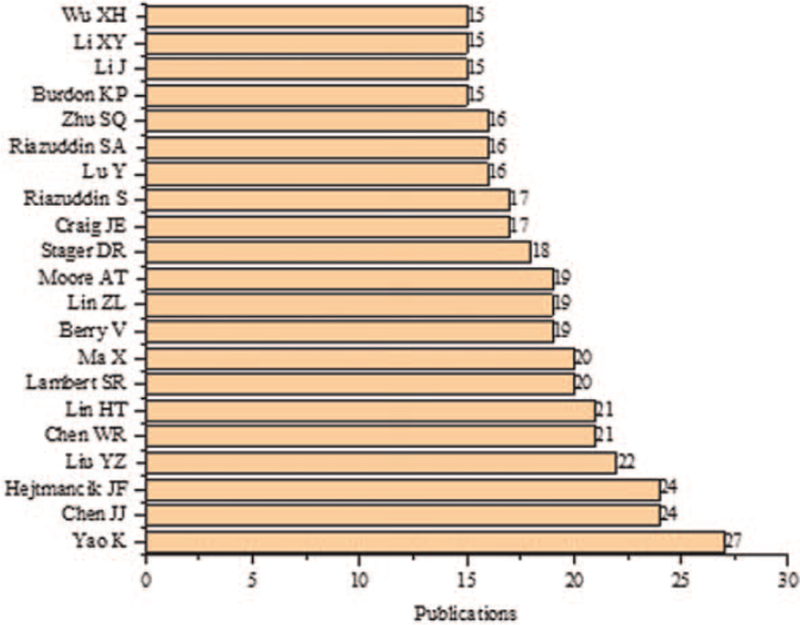
Top authors in congenital cataract with at least 15 publications.

**Figure 8:**
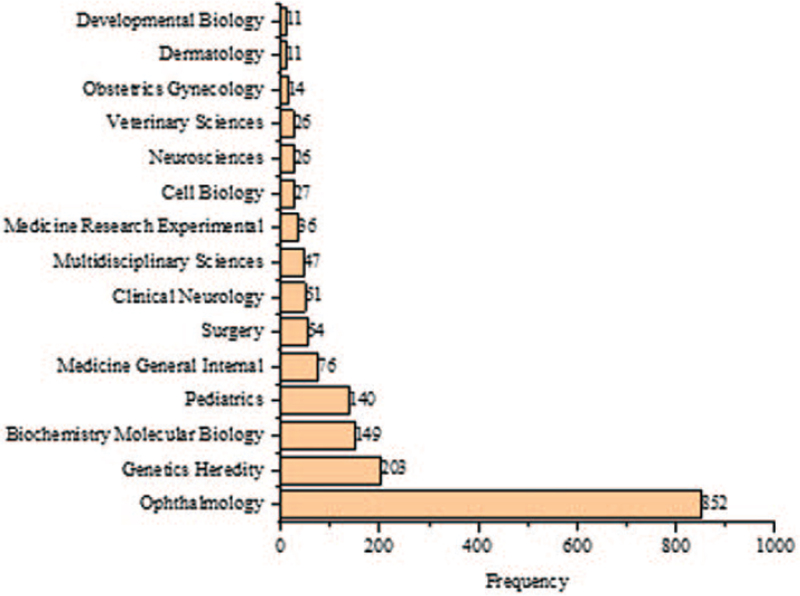
Most studied Web of Science categories in congenital cataract research.

**Figure 9:**
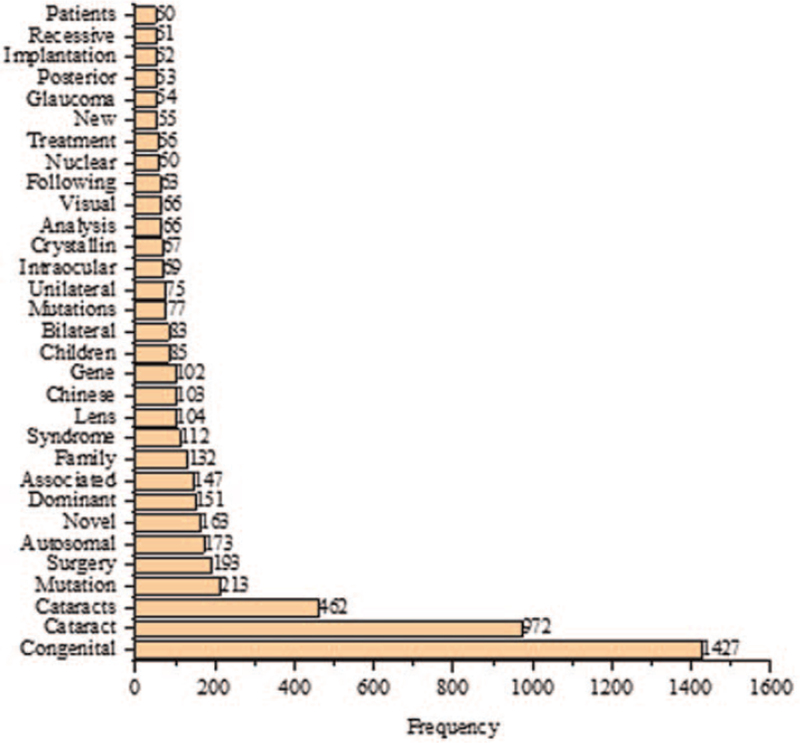
Most frequently used keywords in congenital cataract with at least 50 occurrences.

The most prolific author in cognitional cataracts research was Yao K (n = 27), as shown in Figure 7. The most studied WoS category was ophthalmology (n = 852), as shown in Figure 8. The most widely used keywords were congenital (n = 1427) and cataract (n = 972), as shown in Figure 9. The most cited paper in congenital cataracts was “Congenital cataract following German measles in the mother,” published in *Transactions of the Ophthalmological Society of Australia* in 1941 cited 1268 times, as shown in Table 1.

**Table 1:** Top 10 most cited publications in congenital cataract research.

Ranking	Study reference	Global citations
1	Gregg NM. Congenital cataract following German measles in the mother. Transactions of the Ophthalmological Society of Australia. 1941; 3: 35–46.	1268
2	Glaser T, Jepeal L, Edwards JG, Young SR, Favor J, et al. PAX6 gene dosage effect in a family with congenital cataracts, aniridia, anophthalmia and central-nervous-system defects. Nature Genetics. 1994; 7 (4): 463–471.	545
3	Litt M, Kramer P, LaMorticella DM, Murphey W, Lovrien EW, et al. Autosomal dominant congenital cataract associated with a missense mutation in the human alpha crystallin gene CRYAA. Human Molecular Genetics. 1998; 7 (3): 471–474.	378
4	Li WC, Kuszak JR, Dunn K, Wang RR, Ma WC, et al. Lens epithelial-cell apoptosis appears to be a common cellular basis for non-congenital cataract development in humans and animals. Journal of Cell Biology. 1995; 130 (1): 169–181.	294
5	Hejtmancik JF. Congenital cataracts and their molecular genetics. Seminars in Cell & Developmental Biology. 2008; 19 (2): 134–149.	252
6	Mackay D, Ionides A, Kibar Z, Rouleau G, Berry V, et al. Connexin46 mutations in autosomal dominant congenital cataract. American Journal of Human Genetics. 1999; 64 (5): 1357–1364.	228
7	Beller R, Hoyt CS, Marg E, Odom JV. Good visual function after neonatal surgery for congenital monocular cataracts. American Journal of Ophthalmology. 1981; 91 (5): 559–565.	200
8	Berry V, Francis P, Reddy MA, Collyer D, Vithana E, et al. Alpha-b crystallin gene (CRYAB) mutation causes dominant congenital posterior polar cataract in humans. American Journal of Human Genetics. 2001; 69 (5): 1141–1145.	197
9	Birch EE, Stager DR. The critical period for surgical treatment of dense congenital unilateral cataract. Investigative Ophthalmology & Visual Science. 1996; 37 (8): 1532–1538.	156
10	Renwick JH, Lawler SD. Probable linkage between a congenital cataract locus and the duffy blood group locus. Annals of Human Genetics. 1963; 27 (1): 67–84.	152

### 4.2 Co-authorship countries visualization mapping

**Figure 10:**
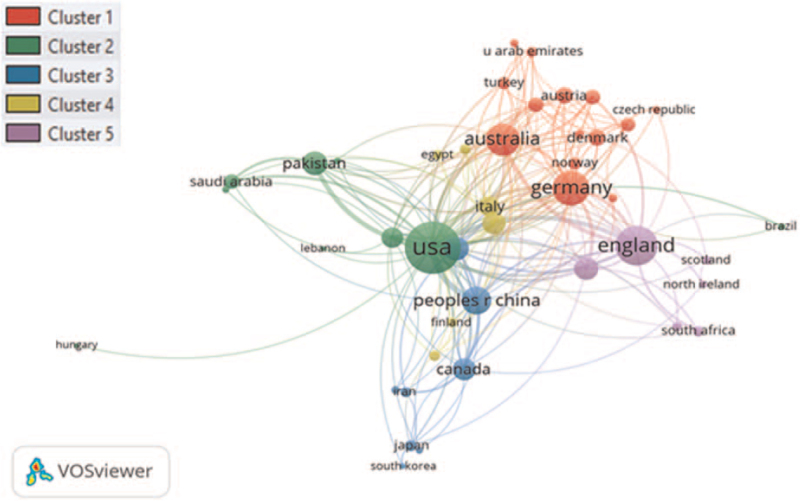
Co-authorship countries visualization network mapping.

The retrieved dataset was plotted for co-authorship visualization network mapping, and the minimum number of publications of a country was fixed at 3. A total of 46 countries were plotted. The USA and England had the highest total link strength (TLS), 189 and 105, respectively, as shown in Figure 10. The minimum cluster size was selected at 5 and the document co-authored by a large number of countries was set at 25. A total of 5 clusters were formed, and each color represents a different cluster. Co-authorship countries overlay visualization mapping is presented in Figure S3, Supplemental Digital Content.

### 4.3 Co-occurrence author keywords visualization mapping

**Figure 11:**
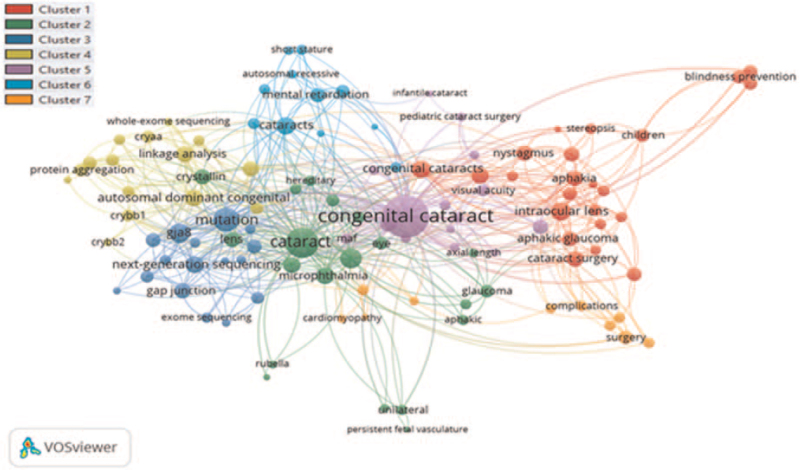
Co-occurrence author keywords visualization network mapping.

Minimum number of occurrences of a keyword was selected at 3. Of the total keywords, only 111 keywords were plotted. Author keywords congenital cataract and cataract had the highest TLS 215 and 98, respectively, as shown in Figure 11. A total of 7 clusters were formed, and each color represents a different cluster. Co-occurrence author keywords overlay visualization mapping is presented in Figure S4, Supplemental Digital Content.

## 5 Discussion

A large number of studies on epidemiology, risk factors, treatment, control, and prevention of congenital cataracts have been conducted by researchers worldwide.^[9,28–34]^ The prevalence of childhood cataracts was found to be 0.42–2.05 and 0.63–13.6 per 10,000 people in low and high-income countries, respectively.^[29]^ Though, these findings do not support the previous reports, the relatively in low-income economies might be due to the low detection rate of cataracts. Population-based epidemiological research is warranted to increase understanding of risk factors and to support the development of novel therapies for childhood cataracts.

However, to the best of our knowledge, no particular bibliometric analysis on congenital cataracts is currently available indexed in the WoSCC database, despite the significant role of bibliometric studies being a referral point for researchers and policymakers, and ophthalmologists. This study focused on analyzing the global research output on congenital cataracts from 1903 to 2021. The study documented the most dynamic authors and countries, most frequent subject areas, most productive journals and authors, and top-cited publications.

In our study, a significant increase in publications on congenital cataracts has been observed over the past few decades. The most studied areas were ophthalmology, genetics heredity, and biochemistry and molecular biology. Like other bibliometric type studies this field was dominated by the USA and other developed countries.^[23,34,35]^

It is unsurprising that the developed countries’ contribution is higher than any other country, as they invest more in scientific research and development.^[33]^ Our results also suggest that collaborations among low-income countries occur much less frequently than collaborations between developed countries. However, serious attention needs to be paid to establish strong research collaboration between low-income countries and developed nations such as the USA.

The current study provides a point of reference for researchers and ophthalmologists besides being a baseline for policymakers and devise effective prevention strategies to combat congenital cataracts. More research is needed to be carried out. The scientists and ophthalmologists from the disease burden countries should be equipped with the latest diagnostic techniques and encouraged to share their findings in peer-reviewed journals.

## 6 Conclusion

These findings provide useful insights into the current status and trends in clinical research in congenital cataracts. The most attractive journal in congenital cataract research was *Investigative Ophthalmology & Visual Science*. The USA was the leading country with the highest publications and TLS. This study might be useful to identify future research domains and provide standard bibliography references for academic and research purposes.

## 7 Limitations

The main limitation of our study is that we used only one database (WoSCC). However, other databases such as PubMed, Scopus, Google Scholar etc, would have provided an additional number of publications and citations on congenital cataracts. The current study limited the search strategy to the title field with document types and publishing language.

## Acknowledgments

The authors acknowledge Southeast University, China, for providing online access to the WoSCC database. The authors also acknowledge their respective institutes and universities.

## Author contributions

**Conceptualization:** Lujain Talaat Idriss, Maryam Hussain, Muhammad Khan, Tauseef Ahmad.

**Data curation:** Maryam Hussain, Tauseef Ahmad.

**Formal analysis:** Tauseef Ahmad.

**Methodology:** Maryam Hussain, Muhammad Khan, Tauseef Ahmad.

**Software:** Tauseef Ahmad.

**Supervision:** Tauseef Ahmad.

**Visualization:** Tauseef Ahmad.

**Writing – original draft:** Maryam Hussain, Muhammad Khan, Tauseef Ahmad.

**Writing – review & editing:** Lujain Talaat Idriss, Maryam Hussain, Muhammad Khan, Tauseef Ahmad, Khushi Muhammad, Mukhtiar Baig, Muhammad Mumtaz Khan, Inamullah.

## Supplementary Material

Supplemental Digital Content

## Supplementary Material

Supplemental Digital Content

## Supplementary Material

Supplemental Digital Content

## Supplementary Material

Supplemental Digital Content
