# Supplementary material for: Mapping of global research output in congenital cataracts from 1903 to 2021
Source: Medicine (Baltimore). 2021 Dec 3;100(48):e27756. doi: 10.1097/MD.0000000000027756 (PMC9191315; doi:10.1097/MD.0000000000027756)

**Supplementary Figure 1** Funding agencies funded at least 10 publications in congenital cataract

**
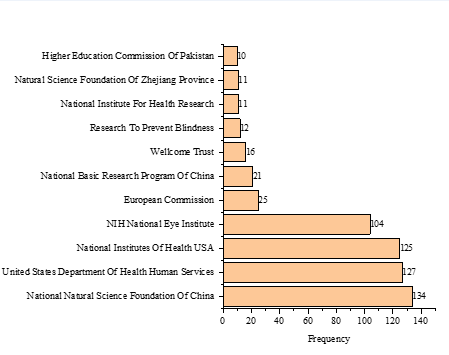
**

**Supplementary Figure 2** Top 15 publishers in congenital cataracts

**
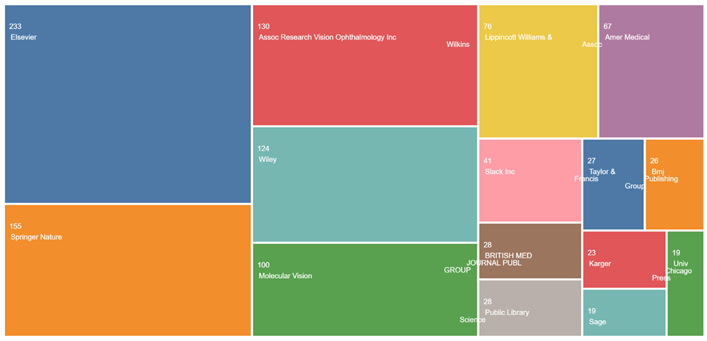
**

**Supplementary Figure 3** Co-authorship countries overlay visualization mapping

**
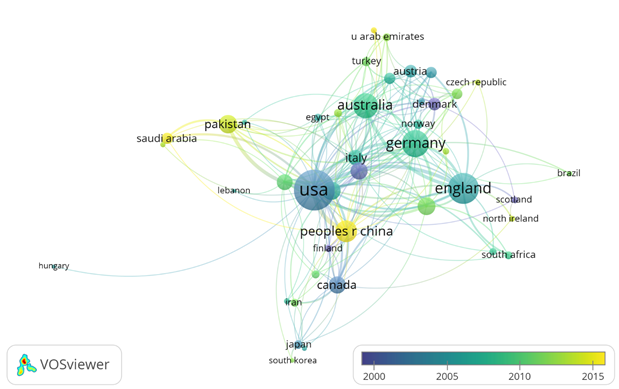
**

**Supplementary Figure 4** Co-occurrence author keywords overlay visualization mapping


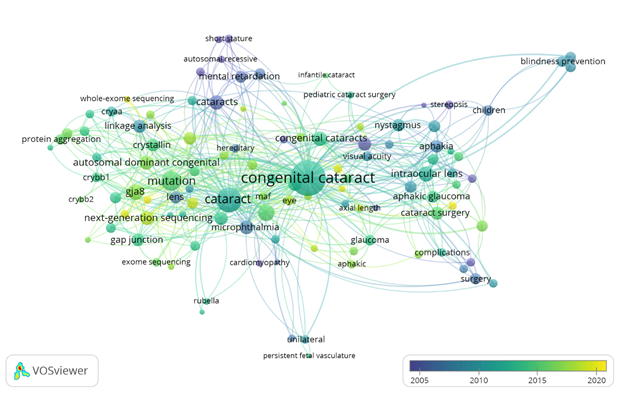

Supplement: Supplemental Digital Content [file medi-100-e27756-s001.doc]
